# Proteomic Analysis of the Secretory Response of *Aspergillus niger* to D-Maltose and D-Xylose

**DOI:** 10.1371/journal.pone.0020865

**Published:** 2011-06-17

**Authors:** José Miguel P. Ferreira de Oliveira, Mark W. J. van Passel, Peter J. Schaap, Leo H. de Graaff

**Affiliations:** Fungal Systems Biology, Laboratory of Systems and Synthetic Biology, Wageningen University, Wageningen, The Netherlands; Louisiana State University, United States of America

## Abstract

Fungi utilize polysaccharide substrates through extracellular digestion catalyzed by secreted enzymes. Thus far, protein secretion by the filamentous fungus *Aspergillus niger* has mainly been studied at the level of individual proteins and by genome and transcriptome analyses. To extend these studies, a complementary proteomics approach was applied with the aim to investigate the changes in secretome and microsomal protein composition resulting from a shift to a high level secretion condition. During growth of *A. niger* on d-sorbitol, small amounts of d-maltose or d-xylose were used as inducers of the extracellular amylolytic and xylanolytic enzymes. Upon induction, protein compositions in the extracellular broth as well as in enriched secretory organelle (microsomal) fractions were analyzed using a shotgun proteomics approach. In total 102 secreted proteins and 1,126 microsomal proteins were identified in this study. Induction by d-maltose or d-xylose resulted in the increase in specific extracellular enzymes, such as glucoamylase A on d-maltose and β-xylosidase D on d-xylose, as well as of microsomal proteins. This reflects the differential expression of selected genes coding for dedicated extracellular enzymes. As expected, the addition of extra d-sorbitol had no effect on the expression of carbohydrate-active enzymes, compared to addition of d-xylose or d-maltose. Furthermore, d-maltose induction caused an increase in microsomal proteins related to translation (e.g., Rpl15) and vesicular transport (e.g., the endosomal-cargo receptor Erv14). Millimolar amounts of the inducers d-maltose and d-xylose are sufficient to cause a direct response in specific protein expression levels. Also, after induction by d-maltose or d-xylose, the induced enzymes were found in microsomes and extracellular. In agreement with our previous findings for d-xylose induction, d-maltose induction leads to recruitment of proteins involved in proteasome-mediated degradation.

## Introduction

Filamentous fungi are remarkable secretors of metabolites and hydrolytic enzymes, which has granted them a prominent role in biotechnology [1]. Many of the hydrolytic enzymes secreted by fungi have evolved to degrade the complex structure of plant cell wall [2]. The most prominent enzymes that are secreted in the process of plant cell wall decomposition are hemicellulases (e.g. xylanases), cellulases, pectinases and proteases. These enzymes have been studied extensively at the biochemical and genetic level due to their wide range of applications. The enzymes are being applied as food and feed additives and are used in the saccharification of plant biomass for the production of bioethanol [3].

In the ascomycete *Aspergillus niger* the biosynthesis of extracellular enzymes is mainly regulated at the transcriptional level. The transcription factors governing expression are either repressors, such as the carbon catabolite repressor CreA [4], or activators such as XlnR and AmyR that turn on the expression of genes encoding (hemi)cellulolytic enzymes in the presence of d-xylose [5] and amylolytic enzymes in the presence of d-maltose [6], respectively. Both the XlnR and the AmyR regulons are repressed by CreA in the presence of d-glucose.

Recent studies have made an effort in improving our understanding of the mechanisms via which extracellular enzymes and proteins in general are secreted in aspergilli. With the publication of the annotated genome of *A. niger* [7], many genes have been identified as functionally related to protein secretion. Based on this annotation and on bioinformatic analyses, another study reported the candidate genes related to protein secretion in *A. niger*, with emphasis on the processes taking place in the endoplasmic reticulum (ER) [8]. In addition to bioinformatic *in silico* predictions of genes related to secretion, the transcriptional responses of *A. niger* were studied under a variety of conditions related to protein secretion, namely by the use of inhibitors of protein glycosylation and protein folding in the ER [9], [10], or by comparison of strains overproducing specific proteins [11]. From these studies it became clear that the unfolded protein response (UPR) together with individual proteins from the secretory pathway play an important role in secretion in *A. niger*. Moreover, the secretome of *A. niger* has been reported in two high-coverage proteomics studies. In one study, *A. niger* was grown on different complex and defined media after which the secretome was compared with the proteins predicted to contain a signal peptide [12]. From this work some 200 secreted proteins were identified. In another study, *A. niger* was grown either in shake-flasks or in a bioreactor, with d-xylose and d-maltose as carbon substrates, and the secretome was compared with the total proteome from cell extracts [13]. Two main conclusions came from this study. First, *A. niger* responds in different ways to the utilization of d-maltose and d-xylose and the spectrum of secreted proteins is very different between shake-flask and bioreactor cultivation. Second, the proteomic response occurs both inside the cell and at the level of secreted enzymes. Despite the above-mentioned studies, the essential mechanisms underlying the high secretion capacity have not been fully elucidated.

In this study we apply a proteomics approach with the aim to investigate potential differences in the secretome and the microsomal proteome of *A. niger* that is grown on sorbitol and that is induced by D-xylose of D-maltose to represent high secretion conditions. While Lu et al. [13] compared the *A. niger* secretome and the intracellular proteome obtained from cell free extracts, this study compares the secretomes and the microsomal proteomes. The low secretion conditions result from fermentor-grown mycelium, using d-sorbitol as a carbon source [14], [15]. Our study reveals significant changes in the microsomal fractions in response to D-xylose and D-maltose induction.

## Results and Discussion

### Secretome and microsomal proteome of *A. niger*

**Figure 1:**
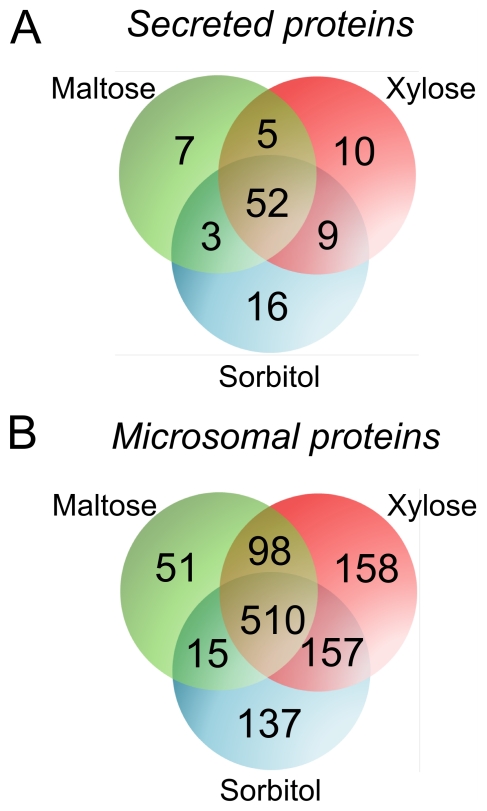
Venn diagrams of the *A. niger* proteins identified. Panel A: Secreted proteins; Panel B: Microsomal proteins. d-maltose, d-xylose, or d-sorbitol was added to *A. niger* cultivated on d-sorbitol.

Analysis of the secreted proteins from the culture filtrates of all three induction conditions using d-sorbitol, d-xylose and d-maltose resulted in the identification of 102 secretome proteins totally. Of these, 52 proteins (51%) are present in all three conditions (Fig. 1A). We did not find any of the major mycelial proteins of aspergilli in the culture filtrate, as described in previous studies [13], [16]. Yet, a few proteins were identified that lacked a recognizable signal sequence or were predicted to be localized intracellular. Since we did not analyze the total intracellular proteome of *A. niger* in this experiment, we cannot exclude the possibility that these proteins were released upon cell lysis and therefore they are presented in a separate table (Additional file 1: Table S1).

We analysed the microsomal proteins after separation by SDS-PAGE and tryptic digestion in gel, which resulted in the identification of 1,126 proteins, of which 510 proteins (45%) were present in all three conditions (Fig. 1B). In *A. niger* approximately 10% of the total proteins are predicted to contain a signal sequence [12]. Signal sequence predictions in our dataset showed that 92% of the secreted proteins contain a signal peptide (Additional file 2: Table S2), whereas 25% of the microsomal proteins contain either a signal peptide or a signal anchor (Additional file 3: Table S3). Microsomal proteins were grouped in the following categories: (i) membrane traffic and protein secretion, 23%; (ii) mitochondrial, 13%; (iii) translation, 12%; (iv) metabolism and defence against reactive oxygen species, 12%; (v) cargo proteins, 8%; (vi) lipid biosynthesis, 8%; (vii) transporters, 5%; and (viii) others or unknown, 14%.

### Secretome under amylolytic and (hemi)cellulolytic conditions

**Figure 2:**
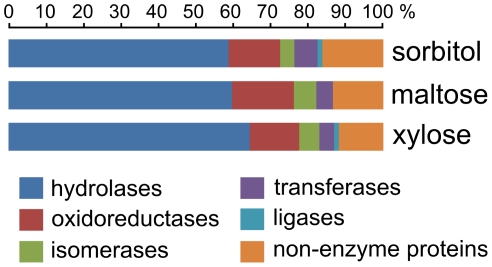
Enzyme class distribution of all secreted proteins. The bar size indicates the percentage of enzymes of the total of secreted proteins detected for each condition.

Proteins of the secretome were classified according to their predicted enzyme class (Additional file 2: Table S2), and the class distribution was determined for each condition. According to enzyme class, hydrolases represented about 60% of the secreted proteins, and as such this was the largest group in all three conditions (Fig. 2). Compared to the total number of proteins secreted, induction by d-xylose resulted in the highest percentage of secreted hydrolases. Growth on d-sorbitol gave rise to the highest percentage of non-enzyme secreted proteins, like the ortholog of *Schizosaccharomyces cerevisiae* cell wall protein Psu1, and a protein of unknown function (JGI37529). On the other hand, d-maltose addition was associated with the highest percentage of oxidoreductases identified in the culture filtrate. In a previous study in which *A. niger* was grown on d-maltose, it was observed that a number of oxidoreductases (e.g. catalases) were expressed at higher levels compared to growth on d-xylose [13]. It was hypothesized that growth on d-maltose increased the amount of reactive oxygen species (ROS) formed extracellularly, compared to d-xylose, and this was accompanied by an increase in enzymes related to ROS. Our results are in line with these previously reported observations.

Each of the secreted proteins was also ranked according to a normalized spectral abundance factor (NSAF), which estimates the protein's relative abundance on the basis of the MS spectral counts and predicted protein size. The most abundant secreted proteins for all three experimental conditions used were the *A. niger* anti-fungal protein (ANAFP), the protease aspergillopepsin A (PepA) and the starch-degrading enzyme glucoamylase A (GlaA). ANAFP is a homologue of *Penicillium* anti-fungal protein (PAF) from *Penicillium chrysogenum*. Like PAF, ANAFP inhibits fungal growth and is probably secreted by *A. niger* as a defense against fungal competitors [17]. Additional abundantly secreted proteins include: (i) β-glucanotransferases, responsible for glucan remodelling, (ii) RNase T2, also known as actibind, which can result in arrested cell growth in plants by binding to actin [18], (iii) chloroperoxidase, that has an important role in lignin degradation, (iv) muconate cycloisomerase that has a putative role in the degradation of aromatic compounds [19], and (v) sulfhydryl oxidase that is most likely involved in maintaining redox balance e.g. through oxidation of reduced glutathione [20]. These proteins presumably have specific functions that need to be active constantly, independently of the external carbon sources.

**Figure 3:**
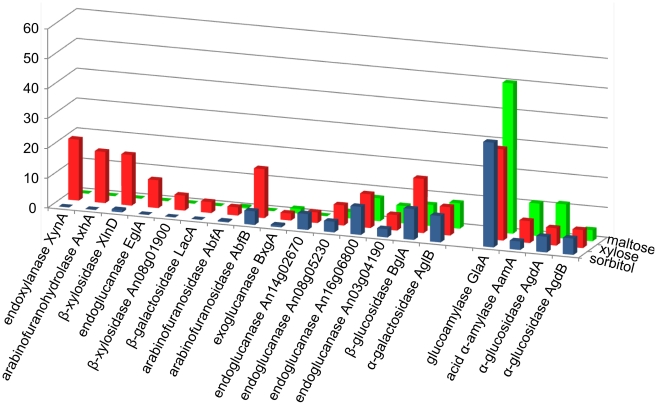
Relative abundance of enzymes related to (hemi)cellulose or starch degradation. Green: d-maltose addition; red: d-xylose addition; blue: d-sorbitol addition.

Following the analysis of highly abundant proteins, the enzymes responsible for starch or (hemi)cellulose degradation were investigated (Fig. 3). The enzymes related to starch degradation were not only present upon d-maltose induction. The starch-degrading enzymes glucoamylase A, acid α-amylase (AamA), and α-glucosidase A (AgdA) were more abundant on the d-maltose condition than on d-xylose or d-sorbitol, whereas α-glucosidase B (AgdB) was found to be less abundant on d-maltose. Although counter-intuitive, this observation may reflect the fact that the expression pattern of *agd*B may differ from that of *gla*A, *aam*A and *agd*A [21], perhaps pointing to a different mode of regulation. In contrast to the amylolytic enzymes that were present even when no d-maltose was added, the enzymes involved in (hemi)cellulose degradation either were present only upon d-xylose induction, or were increased on d-xylose. With regard to d-xylose induction, β-xylosidase XlnD whose gene expression is known to be controlled by XlnR, was detected; however, also a novel putative β-xylosidase (An08g01900) was found that thus far was not reported to be regulated by XlnR. Moreover, proteins previously found to be more highly expressed after d-xylose induction, such as α-xylosidase (AxlA), endoxylanase B, An15g04550 (a putative xylanase), ferulic acid esterase (FaeA), and acetyl xylan esterase (AxeA), were detected upon d-xylose induction. However, the number of unique peptides was insufficient for statistical validation (Additional file 4: Table S4). In addition to this, a clear difference was detected on the relative abundance of different types of galactosidase. The β-galactosidase A (LacA), of which the gene is regulated at the transcriptional level by XlnR, was found more abundant upon d-xylose addition (Fig. 3). On the other hand, α-galactosidase B (AglB) was only present at a very low level. Although the two corresponding genes have previously been shown to be more expressed in the presence of xyloglucan, the *agl*B gene has been found to be also expressed on a large range of substrates [22], in contrast to *lacA*, which is exclusively expressed on xyloglucan-derived substrates. These data support the notion that these galactosidases perform distinct functions in the *A. niger* metabolism. As to the expression of endoglucanases, of the four putative endoglucanases found, only two - An08g05230 and An16g06800 - were more abundant on d-xylose than on the remaining conditions.

**Figure 4:**
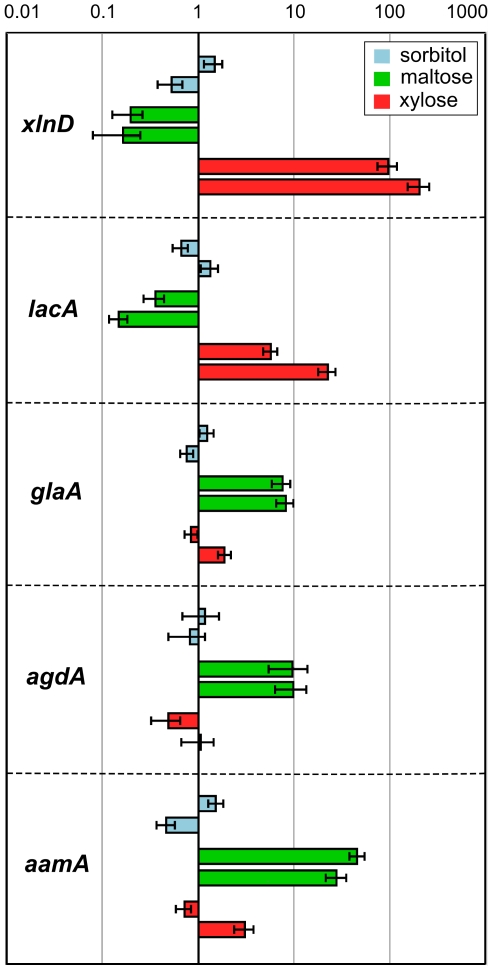
Relative gene expression of selected genes involved in (hemi)cellulose or starch degradation as determined by qPCR. *xlnD*: β-xylosidase D; *lacA*: β-galactosidase A; *glaA*: glucoamylase A; *agdA*: α-glucosidase A; *aamA*: acid α-amylase. Expression values were normalized to the average d-sorbitol values for each gene and are given on a logarithmic scale. Bars represent relative gene expression ± *SE*.

In addition to the proteomic analysis, we investigated the relative gene expression patterns of *xln*D, *lac*A, *gla*A, *agd*A and *aam*A by qPCR analysis. Compared to the d-sorbitol non-inducing condition, the *xln*D and *lac*A genes were highly expressed after d-xylose induction, while *gla*A, *agd*A and *aam*A were highly expressed after d-maltose induction (Fig. 4). The combined results of the secretome and the transcription analyses confirmed that, in this experimental setting, d-maltose and d-xylose induced the expression of specific enzymes and these enzymes were secreted into the extracellular medium.

A G-test [23] was then used as a statistical analysis to assess differential relative abundance for each condition (Table 1), on the basis of individual NSAF values. In our studies, d-maltose induction differentially increased the extracellular amounts of the aforementioned acid α-amylase and of endo-arabinanase AbnC.

**Table 1:** Proteins differentially represented in the secretome.

Protein	Locus tag	G-score* d-maltose	G-score* d-xylose
*Abundant on d-maltose addition only*			
Endo-arabinanase AbnC (p)	An02g10550	5.99	
Acid α-amylase AamA	An11g03340	5.04	
*Abundant on d-xylose addition only*			
Endoxylanase XynA	An03g00940		23.59
Arabinofuranohydrolase AxhA	An03g00960		19.29
β-Xylosidase XlnD	An01g09960		18.25
Endoglucanase A EglA	An14g02760		8.23
Arabinofuranosidase B AbfB	An15g02300		7.13
Cell wall protein PhiA (p)	An14g01820		5.27
β-Xylosidase (p)	An08g01900		4.97
*Lower on d-maltose addition only*			
Unknown hypothetical	37529	8.27	
Antifungal protein ANAFP	An07g01320	7.06	
Amidase (p)	An05g01860	4.26	
*Lower on d-xylose addition only*			
Guanyl-specific RNase T1 (p)	42238		8.61
*Lower on d-maltose and d-xylose addition*			
RNase T2 (actibind)	An01g10580	4.16	13.70
Sensor of PKC1-MPK1 Wsc1 (p)	An03g00250	4.24	5.10

*G-score values of ≥3.841 correspond to P<0,05 and G-score values ≥10.828 to P<0,001 ( http://www.itl.nist.gov/div898/handbook/eda/section3/eda3674.htm **).**

All proteins contain a predicted signal peptide. (p): putative function, identity inferred from sequence similarity.

For the d-xylose condition, together with the above-mentioned enzymes for (hemi)cellulose degradation, the homologue of *Aspergillus fumigatus* PhiA appeared to be over-represented. PhiA is a cell surface protein essential for phialide and conidium-spore development. These results suggest that d-xylose may have a positive effect on PhiA expression at the cell surface in *A. niger*. PhiA has been previously found to have its gene expression increased in an XlnR-overexpressing strain of *A. oryzae* grown on d-xylose as carbon substrate [24]. This suggests that in *A. niger* this protein may be over-expressed on d-xylose through the action of XlnR, although the rationale of the apparent link is not obvious.

The secreted proteins that are less abundant on d-maltose, on d-xylose or both of these conditions include enzymes such as RNases, amidases, ANAFP and a homologue of yeast Wsc1 (Slg1) protein, involved in cell wall maintenance (Table 1). The reason why these secreted proteins are decreased upon d-maltose or d-xylose induction is unclear. Possibly, these proteins are not subject to regulation by carbon substrate, and their decreased abundance relative to the total proteins secreted is a consequence of increased production of the extracellular enzymes induced by d-xylose or d-maltose.

### Comparative analysis of the microsomal proteome

In one of our previous studies, the effect of induction by d-xylose on microsomal proteins of *A. niger* was investigated using the same experimental setup as was used in the current study [15]. Here, we have studied the induction by d-maltose and made a comparison with the d-xylose induced conditions. For the comparison of the different microsomal proteins present in each condition, the same method as the one used for secretome analysis was followed, i.e. ranking of the differential relative abundance by a G-test. An extensive list of proteins was obtained (Tables 2 and 3), and only the proteins with a G-score larger than 10 are discussed, i.e., the top 25% of the significantly increased and the top 10% of the decreased proteins upon induction.

**Table 2:** Protein homologues of the microsomal proteins significantly increased in the d-maltose and d-xylose conditions.

Category	More abundant on d-maltose and d-xylose	More abundant on d-maltose only	More abundant on d-xylose only
**Biosynthetic cargo**	AamA (13.1/5.8), *Sc*Lap4 (4.2/4.4)	GlaA (16.8)	XlnD (17.3), AxlA (6.6)
**Translation-related**	*Sc*Rpp0 (11.3/4.2)	*Sc*Rpl15 (16.1), *Sp*vip1 (5.9), *Sc*Rpl31 (5.8), *Sc*Rps31 (5.8), *Sc*Rps6 (4.7), *Sc*Rpl1 (4.7), *Sc*Pth2 (4.2)	*Sp*Cdc60 (5.8)
**Cytosolic chaperones**		*Sc*Zuo1 (3.9)	
**Translocation to the ER**		*Sc*Sec11 (8.5)	
**Protein anchoring**			*Sc*Gpi12 (9.5)
**ERAD**	*Sc*Pre7 (25.0/26.2); *Sc*Pre5 (18.3/16.8), *Sc*Cdc48 (10.9/20.5); *Sc*Pre6 (8.5/11.9), *Sc*Pre9 (10.0/9.9), *Sc*Scl1 (9.8/9.2), *Sc*Pre8 (10.5/8.4), *Sc*Pup2 (10.4/7.5), *Sc*Pre10 (7.5/8.5), *Sc*Cep52 (10.4/7.5)	*Sc*Pre1 (4.7), *Sc*Pup1 (4.5)	
**Vesicular transport**	RhoA (16.6/8.6), SrgA (8.5/5.7), *Hs*RAB5C (5.6/6.4)	*Sc*Erv14 (20.5), *Sc*Yip1 (10.1), *Hs*ARF6 (8.0), *Sc*Vps21-like protein (5.8), *Sc*Sec17 (4.8)	
**Golgi/post-Golgi sorting**		*Sc*Vps68-like (4.1)	
**Lipid biosynthesis and CYP450 enzymes**		*Ao*PLTPAO (8.5), *Ap*OrdA (4.3)	*Af*AFUA_1G10100 (6.1)
**Mitochondrial**	*Nc*nuo-14 (5.5/13.3), *Sc*Cox4 (8.0/8.4), *Nc*nuo-51 (4.5/11.1), *Sp*qcr1 (4.6/11.0), *Sc*Aco1 (5.2/6.5), *Hs*SLC25A13 (4.1/7.2), *Sc*Yhm1 (4.2/6.7), *Sc*Hsp60 (4.0/4.7)	*Sc*Qcr8 (15.4), *Nc*nuo-12.3 (6.8), *Nc*nuo-19.3 (6.2), *Sp*SPAPJ691.03 (5.3), *Sc*Atp3 (4.2), *Sc*Pic2-like (3.9)	*Sc*Mcr1 (7.5), *Sc*Yhm2 (6.7), *Nc*CYT-1 (6.2), *Sp*rip1 (5.7), *Sc*Atp7 (5.5), *Yl*:YALI0F25135p (5.4), *Nc*nuo-78 (4.8), *Hs*NAA16 (4.7), *Sc*Fcj1-like (4.5), *Sc*Phb1(4.1), *Sc*Ctp1 (4.0), *Nc*nuo-21.3 (3.8)
**Transporters**	*And*PmaA (14.1/11.8)		
**Metabolism**	OahA (14.6/14.2)	*Hs*CHDH (6.9), *Sc*Eno1 (6.6), TpiA (6.3), An02g10150 (6.0), *Sc*Gln1 (4.7)	XyrA (14.6), *Sc*Dld1 (5.8), *Af*Gcy1 (4.7)
**Nuclear**	*Sp*Smd1 (4.0/4.5)	*Sc*Hhf1 (19.5), *Sc*Nop1 (5.0)	
**Unknown and others**		An04g08060 (9.6), An04g05750 (8.3), *At*NIT1 (6.5), *Sc*Env7 (4.5)	*Af*AFUA_3G08290 (5.4), An11g07020 (4.9), An11g00890 (4.7), *Af*AFUA_4G03280 (4.4), *Sc*YHR045w (4.1), An18g01000 (4.0)

*Af: A. fumigatus; And: A. nidulans; Ao: A. oryzae; Ap: A. parasiticus; At: Arabidopsis thaliana; Hs: Homo sapiens; Nc: Neurospora crassa; Sc: Saccharomyces cerevisiae; Sp: Schizosaccharomyces pombe; Yl: Yarrowia lipolytica*. Values in parentheses: G-scores for differential presence.

**Table 3:** Protein homologues of the microsomal proteins significantly decreased in the d-maltose and d-xylose conditions.

Category	Less abundant on d-maltose and d-xylose	Less abundant on d-maltose only	Less abundant on d-xylose only
**Biosynthetic cargo**		An05g02280 (4.5)	
**Translation-related**	*Sc*Wrs1 (7.9/5.7)	*Sc*Rps16b (8.9), *Hs*Rps24 (4.9)	*Sc*Rpl17b (7.4), *Sc*Rpl27 (7.1), *Hs*Rps18 (6.5), *Sc*Rpl28 (6.3), *Sc*Rps9 (5.1), *Sc*Rpl21A (5.1), *Sc*Rps20 (4.6), *Sc*Rpl7A (4.4), *Hs*Rpl12 (4.0), *Sc*Rpl3 (3.9)
**Cytosolic chaperones**	CypA (14.2/10.9)	SspB (5.0)	*Hs*HSPA8 (9.4)
**Translocation to the ER**			*Sc*Sbh2 (10.0), *Sc*Srp102 (5.4)
**Protein glycosylation/QC**		AgdE (6.8)	
**Vesicular transport**	*Ao*AO090026000708 (9.0/6.0)	*Sc*Yop1 (6.5), *Sc*Sec21 (6.1)	
**Golgi/post-Golgi sorting**	*Hs*CLTC (9.8/5.9)		
**Morphogenesis and cytoskeleton**	*And*HEX1 (9.1/6.3)	*Sp*cdc4 (9.5), *Sp*SPBC31F10.16 (5.3), *And*MpkA (4.0)	*Sc*Sac6 (4.1)
**Lipid biosynthesis and CYP450 enzymes**	*Sc*Sec14 (4.2/10.2), *Sp*fas2 (4.7/4.2)	*Sc*Tsc10 (5.8), AclA (4.8)	
**Mitochondrial**		*Sc*Idh2 (4.4), *Hs*GOT2 (4.4)	*Sc*Lat1 (4.7)
**Transporters**		*Hs*KCNAB2 (4.0)	
**Metabolism**	*Sc*Gcy1 (10.5/9.0), ArgB (11.3/7.9), *Sc*Ser3 (11.1/6.3), *Sc*Hnt1 (4.1/7.5)	*Sc*Adk1 (4.7), *Sc*Aat2 (4.6), *Hs*PYCR1 (4.5), *Sc*Gph1 (4.2)	*Ao*AO090020000635 (5.2), *Sp*SPAC19G12.04 (4.3), *Sc*Cys4 (4.1)
**ROS defence**	An02g12940 (5.1/4.0)		
**Nuclear**		*Sc*Nic96 (6.5), An09g00500 (4.6), *Sc*Nup170 (4.0)	
**Unknown and others**	*Hs*CIRBP (5.5/9.4)	*Nc*NCU03370 (10.7), *Sp*SPCC1450.12 (3.9)	An12g05040 (7.9), An18g00950 (7.8)

*And: Aspergillus nidulans; Ao: Aspergillus oryzae; Hs: Homo sapiens; Nc: Neurospora crassa; Sc: Saccharomyces cerevisiae; Sp: Schizosaccharomyces pombe*. Values in parentheses: G-scores for differential presence.

The proteins overrepresented upon d-maltose or d-xylose induction are summarized in Table 2. In total, 27 proteins were significantly more abundant upon d-maltose or d-xylose induction as compared to the d-sorbitol control. On d-xylose 26 proteins were more abundant and on d-maltose 37 proteins. The proteins that were more abundant on the d-maltose and d-xylose conditions compared to d-sorbitol were identified as ERAD or mitochondrial proteins. In previous work we have shown that the proteasome 20S core particle is associated with the microsomal fractions by recruitment upon d-xylose induction, but this does not occur upon d-sorbitol addition [15]. Here we find identical results upon induction with d-maltose. The 20S proteasome assembly is an ATP-dependent process, and the increased presence of mitochondrial proteins suggests that upon d-maltose and d-xylose induction a larger fraction of the mitochondria are associated with the ER, thereby facilitating the export of ATP and consequently the 20S proteasome assembly.

Other proteins that are highly expressed in both induction conditions include the ribosomal assembly protein (Rpp0), a small GTPase for vesicular transport (RhoA), a plasma membrane H^+^-ATPase for cell polarity (PmaA) and the metabolic enzyme oxaloacetate acetyl hydrolase involved in oxalate formation [25], [26].

As for the remainder of the proteins, several cargo proteins were specific for one or both inducing conditions. Glucoamylase A was found more abundant on d-maltose, β-xylosidase XlnD was only detected on d-xylose, and acid α-amylase AamA was found on both conditions, though more abundant on d-maltose compared to d-xylose. d-Maltose induction also increased endosomal-cargo receptor Erv14, histone H4 and the protein component of the large subunit Rpl15.

Several proteins were found to be induced by d-maltose or d-xylose, however, only a few proteins were found in decreased amounts under these conditions. The main microsomal proteins that were repressed upon the d-maltose and d-xylose conditions were the cytoplasmic chaperone CypA, the phosphatidylinositol-phosphatidylcholine transfer Sec14 protein and the three metabolic enzymes glycerol dehydrogenase, 3-phosphoglycerate dehydrogenase and ornithine carbamoyl-transferase. In addition, an unknown protein from the perilipin (lipid binding) family was decreased on d-maltose.

The comparative analysis of the secretome and the microsomal proteome reveals that the 29 cargo proteins are both present in the secretory organelles as well as secreted outside of the cell, whereas the anchored secreted proteins are predominantly found in the microsomal fractions (Table 4). Although the secreted proteins GlaA, AamA, AgdA, XlnD, LacA and BglA etc are equally abundant in the microsomal and the secreted fractions, the anchored proteins do not appear in both fractions equally. Also the comparison of protein relative amounts present in the secretome and in microsomes is also difficult because of the factor time, since due to the experimental set up, the secreted proteins accumulated over a period of 16 h. The microsomal proteome on the other hand was the result of microsomes isolated in a defined moment in time; therefore some proteins might accumulate whereas others might be immediately secreted.

**Table 4:** Proteins shared by the secretome and microsomal proteome.

Protein	Locus tag	Microsomal(NSAF.10^4^)			Secreted (NSAF.10^3^)		
		Mal.	Xyl.	Sorb.	Mal.	Xyl.	Sorb.
Glucoamylase GlaA	An03g06550	63.9	41.3	25.8	50.4	30.6	35.1
Acid α-amylase AamA	An11g03340	14.6	8.4	1.3	10.9	7.4	2.9
α-Glucosidase AgdA	An04g06920	5.5	3.0	1.5	11.5	5.8	5.2
β-Xylosidase XlnD	An01g09960	0.1	13.6	0.2	0.4	17.0	0.8
β-Galactosidase LacA	An01g12150	0.1	2.2	0.4	0.3	3.7	0.2
β-Glucosidase BglA/bgl1	An18g03570	3.1	9.5	5.6	7.1	18.2	10.3
Glucanotransferase BgtB	An03g05290	3.0	4.1	4.2	25.7	35.8	23.5
Glucanotransferase GelA	An10g00400	5.4	5.7	2.0	23.5	22.2	34.7
Glucanotransferase GelD	An09g00670	2.6	3.2	4.1	9.1	7.8	9.5
Glucanotransferase	53033	3.8	6.2	2.7	10.3	10.6	8.8
CW protein CrhD	An01g11010	4.6	6.1	2.3	8.1	5.6	7.8
Serine-type carboxypeptidase I	An02g04690	0.6	1.2	0.2	7.2	5.2	9.0
Carboxypeptidase S1	An03g05200	2.5	4.6	5.7	18.0	14.5	12.7
Barrierpepsin	An18g01320	1.2	2.6	2.0	3.2	3.8	4.1
CW organization protein EcmA	An04g01230	2.4	4.3	2.9	12.3	14.2	23.1
Catalase CatR	An01g01820	9.8	15.4	10.9	9.2	6.5	10.4
Monophenol monooxigenase	An01g09220	4.5	2.9	4.2	19.3	16.5	16.8
Muconate cycloisomerase	An01g14730	1.4	3.5	5.0	24.5	27.3	23.7
Hydroxynicotine oxidase	An07g02360	6.0	6.7	7.8	23.7	21.2	22.8
Conserved hypothetical	An04g08730	1.0	1.2	3.2	8.1	9.7	10.4
Adenosine permease	An10g00800	1.8	0.7	2.9	13.6	5.5	7.6

CW: cell wall; SP: signal peptide; NSAF: normalized spectral abundance factor; Mal.: d-maltose; Xyl.: d-xylose; Sorb.: d-sorbitol. Underlined values: NSAF increased compared to d-sorbitol control. Values in parentheses: NSAF decreased compared to d-sorbitol control.

### Conclusions

A proteomic approach was used to analyze the changes in the secretome and the microsomal proteome of *A. niger* after induction of extracellular enzyme production. Using a semi-quantitative method based on MS spectral counts, we were able to estimate the relative amounts of secreted and secretory proteins upon induction of extracellular enzyme production. This study confirms that d-maltose and d-xylose each induce the production of specific extracellular enzymes in *A. niger*. Moreover, we were able to show that induction by d-maltose also results in the association of the 20S core of the proteasome with secretory organelles, similar to what has been recently reported for d-xylose induction. This suggests that the recruitment of the proteasome may be a general feature of the shift to a secretion state of the cell. Our study shows the power of shotgun proteomic analysis of enriched cell organelle fractions in the study of protein secretion in filamentous fungi and will contribute to understanding the underlying mechanisms involved in the high secretion capacity of these organisms.

## Methods

### Strain and culture conditions

All experiments were conducted using the *A. niger* wild type strain N400 (CBS 120.49), cultured in 2.5 liters fed-batch bioreactors (Applikon). For pre-cultures 1.0×10^6^ spores per milliliter were inoculated in 2.2 liters of minimal medium [27] containing 0.05% yeast extract and 100 mM d-sorbitol, at 30°C. Spore germination was as described previously [14], with headspace aeration and a stirring speed of 300 rpm. When dissolved oxygen levels decreased to 60%, stirring speed was switched to 750 rpm and the sparger inlet was used for aeration. This moment was considered the actual culture starting point. After 14 h from the culture starting point, d-maltose, d-xylose or d-sorbitol (control) was added to each culture (10 mM final concentration) and 2 hours later a 100 ml sample was taken from each fermentor and filtered through nylon gauze. Part of the harvested mycelium was processed for subcellular fractionation and the other part was immediately frozen in liquid nitrogen and stored at −80°C. Culture filtrate was also frozen in liquid nitrogen and stored at −80°C. All experiments were done as two biological replicates.

### Sample preparation for LC-MS/MS

Microsome enrichment techniques and subsequent preparation of proteins for LC-MS/MS were as described previously [15]. Parallel to this, culture filtrate aliquots from each condition were concentrated using Microcon YM-10 columns (cut-off 10 kDa, Millipore, Eschborn, Germany), the final sample contained at least 100 µg protein as determined by the Bradford method [28] and was processed for LC-MS/MS analysis. Concentrated protein samples were reduced with DTT and alkylated with iodoacetamide. Afterwards, excess iodoacetamide was removed by addition of cysteine (70 mM final concentration). Samples were overnight digested with 10 ng/µl trypsin (sequencing grade modified trypsin, Promega, Madison, WI, USA) at room temperature, and after this, trifluoroacetic acid was added to a final pH of 2.5.

### LC-MS/MS analysis and protein identification

LC-MS/MS was performed as described previously [29]. In summary, each sample was loaded on a pre-concentration column and the respective peptides were passed through an acetonitrile gradient in an analytical column of fixed formic acid concentration. The eluent was subjected to an electrospray potential by means of a coupled platinum electrode. MS spectra were measured in an LTQ-Orbitrap instrument (Thermo Electron, San Jose, CA, USA). MS scans of the four most abundant peaks were recorded in data-dependent mode. The data are available in the PRIDE database [30] (www.ebi.ac.uk/pride) under accession numbers 15312 and 15313. Quantitative MS/MS spectra were searched using the Open Mass Spectrometry Search Algorithm (OMSSA) [31]. The spectra were independently searched against peptide databases derived from the predicted proteomes of *A. niger* strains CBS713.88 and ATCC 1015, and a decoy reverse database constructed from the reverse proteome of strain CBS713.88. All OMSSA searches used the following parameters: a precursor ion tolerance of 0.2 Da, a fragment ion tolerance of 0.3 Da, a missed cleavage allowance of up to and including 2, fixed carbamide methylation, variable oxidation of methionine and deamination of glutamine and asparagine. The E value threshold was determined iteratively from the false discovery rate (FDR) and was set to 0.01, in which case any given FDR is expected to be below 5%. Peptide-spectrum matches with each individual peptide database were ranked by their E-value for each identified spectrum with a threshold E-value<0.01 and the top hit identified peptide sequence was selected. For FDR calculation, top hit spectral matches to peptides in the reversed database were taken and the number of false positives was divided by the number of total positives. Proteins were only considered present under each condition, *viz.* addition of d-maltose, d-xylose or d-sorbitol, whenever at least 2 unique peptides were identified in one of the biological replicates and 1 unique peptide was identified in the other biological replicate for the same condition.

### Relative protein abundance and functional annotation

Relative abundance of each protein within the total pool of proteins was estimated by calculation of normalized spectral abundance factors [32]:
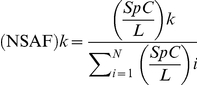
In which (NSAF)_k_ is the normalized spectral abundance factor for a protein *k*, SpC is the number of spectral counts for a given protein plus a pseudocount factor of 0.5, and L is the protein's length. The pseudocount was introduced to enable statistical analyses in the cases where a protein could not be detected in at least one of the three conditions (addition of d-maltose, d-xylose or d-sorbitol). Significant differences in NSAF values were investigated by applying the likelihood ratio G-test for independence with the null hypothesis of equal protein distribution between conditions [33], [34], [35]. Transmembrane domains (TMD) were assessed using the TMHMM tool [36] and signal sequence predictions were done with a local implementation of SignalP 3.0 [37]. Culture filtrate proteins were identified as extracellular enzymes whenever they contained at least one conserved catalytic domain, as assessed by similarity search using blastp and conserved domain search (NCBI). Microsomal proteins predicted were grouped in functional annotation groups guided by previously published functional classification schemes [8], the Functional Catalogue (FunCat) annotation scheme and the predicted molecular function as provided with the *A. niger* CBS 531.88 genome annotation [7].

### Quantitative real-time PCR (qPCR)

All steps from RNA isolation and cDNA synthesis to qPCR and data analysis were as described elsewhere [38]. Briefly, total RNA was extracted, mixed with an exogenous RNA reference transcript, and cDNA was synthesised using Oligo(dT) primers. QPCR SYBR Green Mix (ABgene, Epsom, UK) and specific oligonucleotide primers (Additional file 5: Table S5) were used for qPCR. Two independent PCR runs were performed per cDNA sample from each biological replicate. The Pfaffl method [23] was used to calculate relative gene expression levels, double-normalized to the added exogenous RNA transcript and to the standard condition, i.e. the average normalized expression of the two biological samples from the d-sorbitol control condition.

## Supporting Information

Table S1 **Predicted intracellular proteins identified from the culture filtrates.**
(XLS)

Table S2 **Secreted proteins identified from the culture filtrates.**
(XLS)

Table S3 **Proteins identified from enriched microsomes.**
(XLS)

Table S4 **Lower confidence secreted proteins identified (less than 2 peptides in one biological replicate and 1 in the other).**
(XLS)

Table S5 **Oligonucleotide primers used for QPCR.**
(XLS)
